# Follicle-stimulating hormone and blood lead levels with bone mineral density and the risk of fractures in pre- and postmenopausal women

**DOI:** 10.3389/fendo.2022.1054048

**Published:** 2022-12-20

**Authors:** Huixin Tong, Bo Su, Zhize Liu, Yongjie Chen

**Affiliations:** ^1^ Department of Endocrinology, General Hospital of Northern Theater Command, Shenyang, China; ^2^ Department of Orthopedics, The Second Affiliated Hospital of Harbin Medical University, Harbin, China; ^3^ Department of Orthopedics, Dalian Municipal Central Hospital, Dalian, China; ^4^ Department of Epidemiology and Statistics, School of Public Health, Tianjin Medical University, Tianjin, China

**Keywords:** follicle-stimulating hormone, blood lead levels, fractures – bone, menopause, bone mineral density

## Abstract

**Background:**

The conclusions on the associations of serum follicle-stimulating hormone (FSH) and blood lead levels with bone mineral density (BMD) were controversial. Furthermore, little was known on the impacts of co-existence of serum FSH and blood lead levels on BMD and the risk of fractures in premenopausal and postmenopausal women. Therefore, the present study aimed to examine the associations of serum FSH and blood lead levels with BMD and the risk of fractures in premenopausal and postmenopausal women.

**Methods:**

Data were derived from the National Health and Nutrition Examination Survey. FSH is assayed using the Microparticle Enzyme Immunoassay technology. Blood lead levels were measured using atomic absorption spectrometry. BMD was measured using dual energy X-ray absorptiometry. Fractures were defined as subjects with fractures in any site of hip, wrist, and spine.

**Results:**

This study included 3798 participants. Elevated blood lead levels were associated with increased serum FSH levels (*β*= 48.22, *95% CI*: 40.21~ 56.22). Serum FSH levels were negatively associated with total femur BMD in pre- and postmenopausal women. However, elevated serum FSH levels were associated with a lower lumbar spine BMD and a higher risk of fractures only in postmenopausal women (*β*= -0.0010, *95% CI*: -0.0015~ -0.0006; *OR*: 1.007, *95% CI*: 1.000~1.014, respectively).

**Conclusions:**

Serum lead levels were associated with serum FSH levels. Serum FSH levels were associated with a lower BMD and a higher risk of fractures.

## Introduction

When women are coming to perimenopause, bone mineral density (BMD) will suffer from an accelerated loss with a rate of 1-2% per year (1, 2). The loss rate will be up to 2% per year at post menopause (3, 4). As a result, the risks of osteoporosis and fractures will considerably increase (5–7). Meanwhile, endocrine system will also undergo rapid changes. For example, circulating follicle-stimulating hormone (FSH) levels become elevated but estradiol levels rapidly decline due to ovarian failure as the ability to procreate ceases at menopause (8). Therefore, many studies were conducted to examine the associations of serum FSH and estradiol levels with BMD. However, the conclusions were controversial, especially for FSH (9, 10).

On the other hand, more than 90% of lead stored in bones throughout life. Furthermore, bone tissue becomes a main source of internal exposure lead as a result of the changes in bone turnover at menopause (11). It is documented that lead may be mobilized from the skeleton when bone is demineralized at menopause (12). Therefore, it was speculated that lead exposure might be associated with bone health. However, up to now, the results from different studies were inconsistent (12–14). Furthermore, little was known on the interrelation between serum FSH and blood lead levels and the impacts of their co-existence on BMD and the risk of fractures in premenopausal and postmenopausal women.

Given blood lead levels are continuously decreasing over the past three decades among the US population, it was imperative to re-examine the associations of serum FSH and blood lead levels with BMD (15). Therefore, the objectives of this study were threefold: First, to demonstrate the interrelation between serum FSH and blood lead levels; second, to investigate the impact of co-existence of serum FSH and blood lead levels on BMD; and third, to evaluate the impact of co-existence of serum FSH and blood lead levels on the risk of fractures in premenopausal and postmenopausal women.

## Methods

### Study design

Data analyzed in this study were derived from the third National Health and Nutrition Examination Survey (NHANES III) from 1988 to 1994 and the continuous NHANES from 1999 to 2002. The NHANES is a repeated cross-sectional and random household survey, and aims to assess and supervise the health and nutritional status in the US population. A stratified, multistage random sample was used to create sample from the US population. Since 1959, a series of surveys were conducted in different population groups. To meet emerging needs, the NHANES has become a continuous program since 1999. The NHANES III focused on oversampling many groups, including children aged 2 months to 5 years, older adults aged 60 years or over, Mexican-American persons, and non-Hispanic black persons. However, the continuous NHANES focused on oversampling of low-income group, adolescents aged 12-19 years, older adults aged 60 years or over, African Americans, and Mexican Americans. The response rate was approximately 78%. Mobile examination centers (MECs) were used to conduct health examinations. If participants failed to complete the MECs, an abbreviated health examination was provided. The major information collected in the NHANES include demographic, socioeconomic, dietary, health-related questions, physiological measurements, and laboratory tests.

### Study population

**Figure 1:**
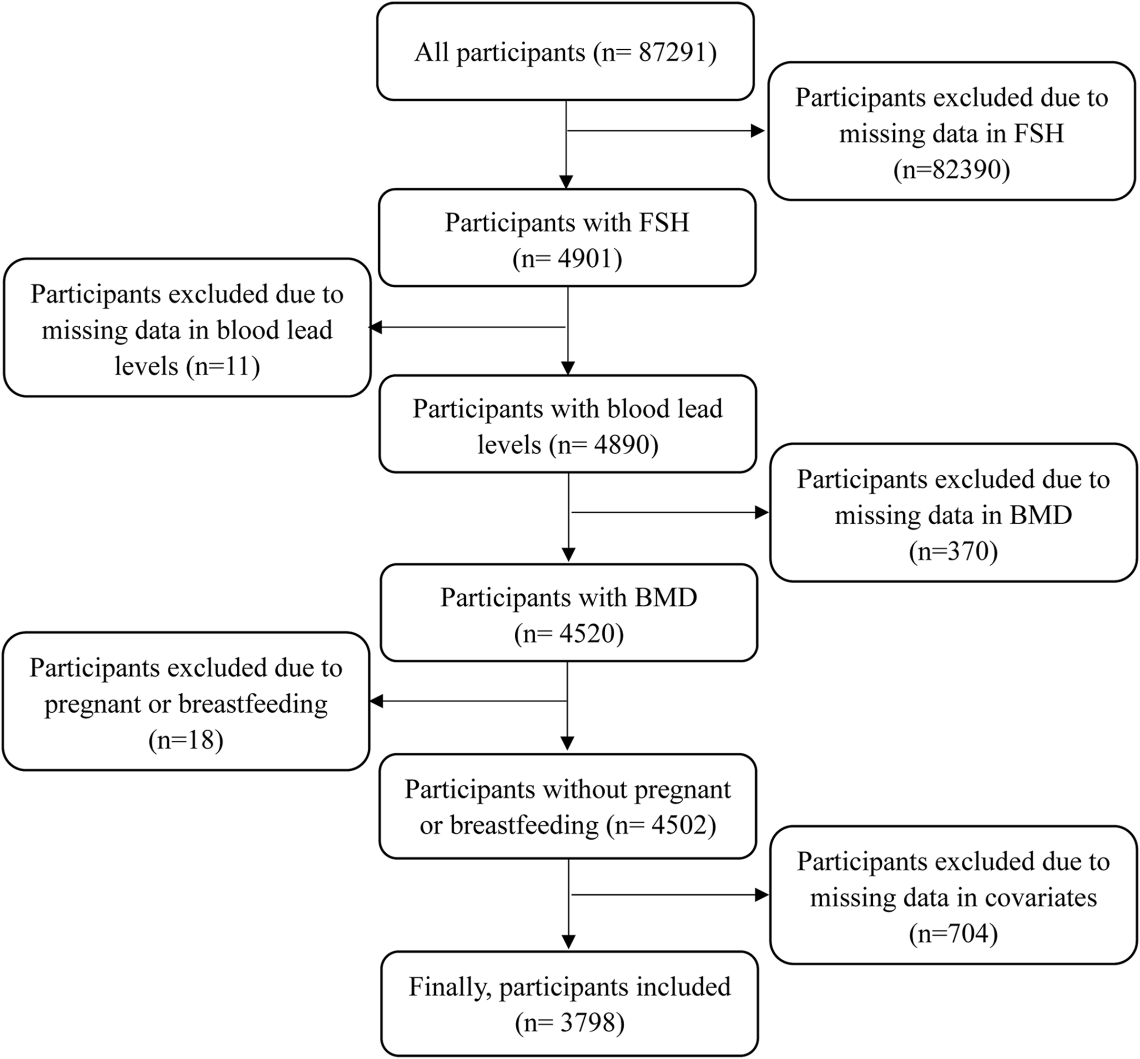
The flow chart of participants inclusion.

This study analyzed all women with complete data in serum FSH levels, blood lead levels, BMD, and the history of fractures. Given a previous study reported that age-related bone loss began as late as 39 years, this study included women with a minimum age of 35 years (16). Meanwhile, the upper limit of age was 60 years as that was the age limit of serum FSH measurement. Besides, women who were pregnant or breastfeeding at the time of survey were excluded. Women with missing data in covariates were excluded. The detailed process is displayed in **Figure 1**. The studies involving human participants were reviewed and approved by the NHANES Institutional Review Board (1999-2002: Protocol #98-12). The participants provided written informed consent to participate in this study.

### Measurements

Fasting blood samples were collected at MECs or at home and were used to assay serum FSH levels, blood lead levels, and other serum biochemical indicators. FSH assay is based on the Microparticle Enzyme Immunoassay (MEIA) technology and was in IU/L. Blood lead levels were measured in umol/L using atomic absorption spectrometry. BMD was measured using dual energy X-ray absorptiometry. Total femur and lumbar spine BMD were collected in the NHANES III and NHANES 1999-2002, respectively. The history of fractures were collected *via* three similar questions: Has a doctor ever told you that you had broken or fractured hip/wrist/spine? If women had fractures in any site of hip, wrist, and spine, she was considered to have a history of fractures.

### Covariates

Age ranged from 35 to 60 years. Height in meter and weight in kilogram were used to calculate body mass index (BMI). Race was classed as non-Hispanic white, non-Hispanic black, Mexican-American, and Other. Current smoking status (yes or no) was identified by a question as follows: Do you now smoke cigarettes/pipe/cigars/use snuff/use chewing tobacco? Current alcohol consumption (yes or no) was identified by a question as follows: Had at least 12 alcohol drinks per year? Alcohol drinks included liquor (such as whiskey or gin), beer, wine, wine coolers, and any other type of alcoholic beverage. Education levels were identified using a question: What is the highest grade or level of school you have completed or the highest degree you have received? Education levels were reclassified as middle school or below, high school, and college or above in the final analysis. Marital status was classified as married, widowed, divorced, separated, and never married. Annual household income was reclassified as < $20,000, $20,000~$45,000, and ≥ $45,000. History of hypertension and diabetes, ever been pregnant, had at least one ovary removed, use of hormone therapy, and ever treated for osteoporosis were collected at MECs. If a woman had not menstruated for 12 months or longer, she was considered as a postmenopausal woman. Otherwise, she was considered as a premenopausal woman.

### Statistical analysis

Quantitative data such as age, BMI, and BMD were expressed as means ± standard deviations, and compared between premenopausal and postmenopausal groups using *t* test. Serum FSH levels were expressed as *P_50_* (*P_25_*, *P_75_*) and blood lead levels were expressed as geometric mean (*95% CI*), which were compared between-groups using *Wilcoxon rank sum* test. Qualitative data were expressed as frequencies (percentages) and compared between-groups using *chi-square* test. General linear regression model was employed to analyze the association of blood lead levels with serum FSH levels and the associations of serum FSH and blood lead levels with BMD. Logistic regression model was used to examine the associations of serum FSH and blood lead levels with the risk of fractures and obtain odd ratios (*ORs*) and 95% confidential intervals (95% *CIs*). Furthermore, both general linear regression and logistic regression models were stratified by menopausal status. In adjusted models, covariates such as age, BMI, race, current smoking, current alcohol consumption, education levels, marital status, annual household income, history of hypertension and diabetes, ever been pregnant, had at least one ovary removed, use of hormone therapy, and ever treated for osteoporosis were adjusted. All analyses were conducted using SAS 9.4 (SAS Institute Inc., Cary, NC, USA.). A two-tailed *P*≤ 0.05 was considered to be statistically significant.

## Results

This study included 3798 participants in the final analysis. There were 2506 participants from the NHANES III and 1292 participants from the NHANES 1999-2002. In total, the average of age was 45.97± 7.41 years. There were 2172 premenopausal and 1626 postmenopausal women. The median of serum FSH levels was 11.27(5.69, 51.00) IU/L. The geometric mean of blood lead levels was 0.095 (0.093, 0.098) umol/L. The averages of total femur and lumbar spine BMD were 0.94± 0. 15 and 1.05± 0.16 g/cm^2^, respectively. There were 190 women with history of fractures, accounting for 5.00%. Furthermore, compared to premenopausal women, postmenopausal women had higher serum FSH and blood lead levels (all *P*< 0.001), lower total femur and lumbar spine BMD (all *P*< 0.001), and an increased rate of fractures (*P*= 0.002). In other covariates, significant differences between-groups were observed in BMI, race, current smoking, current alcohol consumption, education levels, marital status, history of hypertension and diabetes, had at least one ovary removed, use of hormone therapy, and ever treated for osteoporosis, but not in annual household income (*P*= 0.070) and ever been pregnant (*P*= 0.635). The characteristics of all participants are shown in **Table 1**.

**Table 1:** Characteristics of all participants.

Characteristics	Total sample (n=3798)	Premenopausal women (n=2172)	Postmenopausal women (n=1626)	*P*
Age (mean ± SD, years)^※^	45.97± 7.41	41.89 ± 5.07	51.41 ± 6.51	< 0.001
BMI (mean ± SD, kg/m^2^)^※^	28.95± 6.94	28.63 ± 7.08	29.37 ± 6.72	0.001
Race (n (%))^#^				< 0.001
Non-Hispanic white	1714(45.13)	913(42.03)	801(49.26)	
Non-Hispanic black	993(26.15)	575(26.47)	418(25.71)	
Mexican-American	864(22.75)	549(25.28)	315(19.37)	
Other	227(5.98)	135(6.22)	92(5.66)	
Current smoking (n (%))^#^				0.001
No	2877(75.75)	1690(77.81)	1187(73.00)	
Yes	921(24.25)	482(22.19)	439(27.00)	
Current alcohol consumption (n (%))^#^				< 0.001
No	2506(65.98)	1372(63.17)	1134(69.74)	
Yes	1292(34.02)	800(36.83)	492(30.26)	
Education levels (n (%))^#^				< 0.001
Middle school or below	545(14.35)	281(12.94)	264(16.24)	
High school	1786(47.02)	958(44.11)	828(50.92)	
College or above	1467(38.63)	933(42.96)	534(32.84)	
Marital status (n (%))^#^				< 0.001
Married	2500(65.82)	1451(66.80)	1049(64.51)	
Widowed	182(4.79)	46(2.12)	136(8.36)	
Divorced	564(14.85)	304(14.00)	260(15.99)	
Separated	232(6.11)	140(6.45)	92(5.66)	
Never married	320(8.43)	231(10.64)	89(5.47)	
Annual household income (n (%))^#^				0.070
< $20,000	1185(31.2)	646(29.74)	539(33.15)	
$20,000~$45,000	1315(34.62)	761(35.04)	554(34.07)	
≥$45,000	1298(34.18)	765(35.22)	533(32.78)	
History of hypertension (n (%))^#^				< 0.001
No	2444(64.35)	1585(72.97)	859(52.83)	
Yes	1354(35.65)	587(27.03)	767(47.17)	
History of diabetes (n (%))^#^				< 0.001
No	3331(87.70)	1975(90.93)	1356(83.39)	
Yes	467(12.30)	197(9.07)	270(16.61)	
Ever been pregnant (n (%))^#^				0.635
No	294(7.74)	172(7.92)	122(7.50)	
Yes	3504(92.26)	2000(92.08)	1504(92.50)	
Had at least one ovary removed (n (%))^#^				< 0.001
No	3169(83.44)	2094(96.41)	1075(66.11)	
Yes	629(16.56)	78(3.59)	551(33.89)	
Ever treated for osteoporosis (n (%))^#^				< 0.003
No	3755(98.87)	2160(99.45)	1595(93.79)	
Yes	43(1.13)	12(0.55)	31(6.21)	
Use of hormone therapy (n (%))^#^				< 0.001
No	3416(89.94)	2101(96.73)	1315(80.87)	
Yes	382(10.06)	71(3.27)	311(19.13)	
History of fractures (n (%))^#^				0.003
No	3608(95.00)	2083(95.90)	1525(93.79)	
Yes	190(5.00)	89(4.10)	101(7.80)	
FSH (*P_50_* (*P_25_*, *P_75_*), IU/L)^§^	11.27(5.69, 51.00)	6.70(4.65, 11.39)	51.00(22.81, 72.80)	< 0.001
Blood lead levels ((geometric mean (*95% CI*), umol/L)^§^	0.095 (0.093, 0.098)	0.085 (0.083, 0.088)	0.111 (0.108, 0.115)	< 0.001
Total femur BMD^※^ (mean ± SD, g/cm^2^)	0.94± 0.15	0.96 ± 0.14	0.90 ± 0.15	< 0.001
Lumbar spine BMD^※^ (mean ± SD, g/cm^2^)	1.05± 0.16	1.08 ± 0.15	1.02 ± 0.16	< 0.001

BMI, body mass index; FSH, follicle-stimulating hormone; BMD, bone mineral density.

^※^These variables were analyzed using *t-test.*

^#^These variables were analyzed using *chi-square.*

^§^These variables were analyzed using *Wilcoxon rank sum test.*

**Figure 2:**
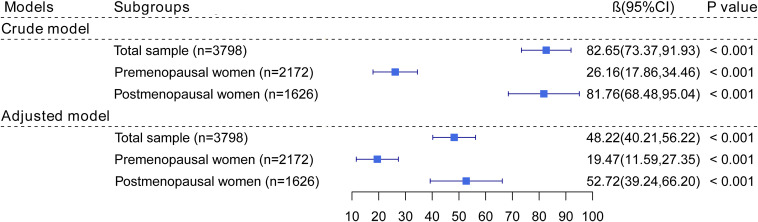
The association of blood lead levels with FSH. In adjusted models, age, BMI, race, current smoking, current alcohol consumption, education levels, marital status, annual household income, history of hypertension and diabetes, ever been pregnant, use of hormone therapy, and had at least one ovary removed were adjusted. In postmenopausal women, years since menopause was additionally adjusted.

**Figure 2** shows the association of blood lead levels with serum FSH levels. Whether adjusting for covariates or not, elevated blood lead levels were associated with increased serum FSH levels (*β*= 48.22, *95% CI*: 40.21~ 56.22, *P*< 0.001). When stratified by menopausal status, there was a positive association of blood lead levels with serum FSH levels in both premenopausal and postmenopausal women (*β*= 19.47, *95% CI*: 11.59~ 27.35, *P*< 0.001; *β*= 52.72, *95% CI*: 39.24~ 66.20, *P*< 0.001, respectively).

In total population, serum FSH levels were negatively associated with total femur and lumbar spine BMD (*β*= -0.0008, *95% CI*: -0.0010~ -0.0006 *P*< 0.001; *β*= -0.0009, *95% CI*: -0.0012~ -0.0006, *P*< 0.001, respectively). However, there were no significant associations of blood lead levels with total femur and lumbar spine BMD (*β*= -0.0364, *95% CI*: -0.0810~ 0.0082, *P*= 0.110; *β*= -0.0945, *95% CI*: -0.2233~ 0.0342, *P*= 0.150, respectively). When serum FSH and blood lead levels were simultaneously analyzed, elevated serum FSH levels but not blood lead levels were associated with lower total femur and lumbar spine BMD (*β*= -0.0008, *95% CI*: -0.0010~ -0.0006, *P*< 0.001; *β*= -0.0009, *95% CI*: -0.0012~ -0.0006, *P*< 0.001, respectively). (**Table 2**)

**Table 2:** The associations of FSH and blood lead levels with bone mineral density^※^.

Models	Total femur bone mineral density			Lumbar spine bone mineral density		
	*β*	*95% CI*	*P*	*β*	*95% CI*	*P*
Model 1						
FSH	-0.0008	-0.0010~ -0.0006	< 0.001	-0.0009	-0.0012~ -0.0006	< 0.001
Model 2						
Blood lead levels	-0.0364	-0.0810~ 0.0082	0.110	-0.0945	-0.2233~ 0.0342	0.150
Model 3						
FSH	-0.0008	-0.0010~ -0.0006	< 0.001	-0.0009	-0.0012~ -0.0006	< 0.001
Blood lead levels	-0.0075	-0.0523~ 0.0373	0.742	0.0013	-0.1306~ 0.1331	0.985

FSH, follicle-stimulating hormone.

^※^In all models, age, BMI, race, current smoking, current alcohol consumption, education levels, marital status, annual household income, history of hypertension, diabetes, and fractures, ever been pregnant, had at least one ovary removed, use of hormone therapy, and ever treated for osteoporosis were adjusted.

**Table 3** shows the associations of serum FSH and blood lead levels with BMD stratified by menopausal status. For total femur BMD, independently of menopausal status, serum FSH levels were negatively associated with total femur BMD when serum FSH and blood lead levels were analyzed separately. Similarly, only serum FSH levels were negatively associated with total femur BMD (premenopausal women: *β*= -0.0007, *95% CI*: -0.0011~ -0.0003, *P* = 0.001; postmenopausal women: *β*= -0.0008, *95% CI*: -0.0011~ -0.0005, *P*< 0.001) when they were simultaneously analyzed. For lumbar spine BMD, serum FSH and blood lead levels were not associated with BMD whether they were analyzed separately or simultaneously in premenopausal women. However, serum FSH levels were negatively associated with BMD in postmenopausal women when it was analyzed separately (*β*= -0.0010, *95% CI*: -0.0015~ -0.0006, *P*< 0.001) and was analyzed together with blood lead levels (*β*= -0.0011, *95% CI*: -0.0015~ -0.0007, *P*< 0.001). No significant relationship between blood lead levels and BMD was observed in any scenario.

**Table 3:** The associations of FSH and blood lead levels with bone mineral density stratified by menopausal status^※^.

Models	Total femur bone mineral density			Lumbar spine bone mineral density		
	*β*	*95% CI*	*P*	*β*	*95% CI*	*P*
Premenopausal women (n=2172)						
Model 1						
FSH	-0.0007	-0.0010~ -0.0003	0.001	0.0001	-0.0005~ 0.0006	0.849
Model 2						
Blood lead levels	0.0045	-0.0602~ 0.0691	0.892	-0.0564	-0.2335~ 0.1207	0.532
Model 3						
FSH	-0.0007	-0.0011~ -0.0003	0.001	0.0001	-0.0005~ 0.0007	0.797
Blood lead levels	0.0158	-0.0490~ 0.0806	0.633	-0.0588	-0.2371~ 0.1194	0.517
Postmenopausal (n=1626)^#^						
Model 1						
FSH	-0.0008	-0.0011~ -0.0006	< 0.001	-0.0010	-0.0015~ -0.0006	< 0.001
Model 2						
Blood lead levels	-0.0697	-0.1325~ -0.0069	0.030	-0.0743	-0.2691~ 0.1205	0.454
Model 3						
FSH	-0.0008	-0.0011~ -0.0005	< 0.001	-0.0011	-0.0015~ -0.0007	< 0.001
Blood lead levels	-0.0402	-0.1027~ 0.0224	0.208	0.0900	-0.1113~ 0.2912	0.380

FSH, follicle-stimulating hormone.

^※^In all models, age, BMI, race, current smoking, current alcohol consumption, education levels, marital status, annual household income, history of hypertension, diabetes, and fractures, ever been pregnant, had at least one ovary removed, use of hormone therapy, and ever treated for osteoporosis were adjusted.

^#^In postmenopausal women, years since menopause was additionally adjusted.

The associations of serum FSH and blood lead levels with the risk of fractures are present in **Table 4**. In total samples, elevated serum FSH levels were associated with a higher risk of fractures (*OR*: 1.007, *95% CI*: 1.001~1.012, *P*= 0.021) as it was analyzed separately. However, blood lead levels were not associated with the risk of fractures (*OR*: 0.599, *95% CI*: 0.132~2.724, *P*= 0.507) as it was analyzed separately. When serum FSH and blood lead levels were simultaneously analyzed, only serum FSH levels were associated with the risk of fractures (*OR*: 1.007, *95% CI*: 1.002~1.013, *P*= 0.012). In premenopausal women, there were no significant associations of serum FSH and blood lead levels with the risk of fractures in any scenario. However, a significant relationship between serum FSH levels and the risk of fractures was observed in postmenopausal women as it was analyzed separately (*OR*: 1.007, *95% CI*: 1.000~1.014, *P*= 0.041) and it was analyzed together with blood lead levels (*OR*: 1.008, *95% CI*: 1.001~1.015, *P*= 0.033).

**Table 4:** The associations of FSH and blood lead levels with the risk of fractures^※^.

Models	Total samples(190/3798)^#^			Premenopausal women (89/2172)^#^			Postmenopausal women (101/1626)^#,‡^		
	*OR*	*95% CI*	*P*	*OR*	*95% CI*	*P*	*OR*	*95% CI*	*P*
Model 1									
FSH	1.007	1.001~1.012	0.021	0.992	0.975~1.009	0.347	1.007	1.000~1.014	0.041
Model 2									
Blood lead levels	0.599	0.132~2.724	0.507	0.282	0.017~4.653	0.376	0.784	0.115~5.338	0.804
Model 3									
FSH	1.007	1.002~1.013	0.012	0.993	0.975~1.010	0.717	1.008	1.001~1.015	0.033
Blood lead levels	0.405	0.080~2.048	0.827	0.325	0.020~5.308	0.430	0.514	0.065~4.090	0.529

FSH, follicle-stimulating hormone.

^※^In all models, age, BMI, race, current smoking, current alcohol consumption, education levels, marital status, annual household income, history of hypertension, diabetes, and fractures, ever been pregnant, had at least one ovary removed, use of hormone therapy, and ever treated for osteoporosis were adjusted.

^#^Indicating the number of fractures/sample size.

^‡^In postmenopausal women, years since menopause was additionally adjusted.

## Discussion

This study examined the relationship between serum FSH and blood lead levels, as well as the associations of serum FSH and blood lead levels with BMD and the risk of fractures. The results implied that elevated blood lead levels were associated with increased serum FSH levels independently of menopausal status. Serum FSH levels were negatively associated with total femur BMD independently of blood lead levels and menopausal status. However, elevated serum FSH levels were associated with a lower lumbar spine BMD and a higher risk of fractures in postmenopausal women but not in premenopausal women. While there was no significant association of blood lead levels with BMD and the risk of fractures.

Previous studies reported that lead exposure was linked to increased risks of spontaneous abortion, reduced sperm count, and poor sperm motility (17). Several studies declared that lead can accumulate in ovarian follicular fluid and restrain the production of progesterone (18, 19). As a response, FSH levels will increase. The potential mechanisms of lead exposure regulating serum FSH levels were as follows: First, lead can act on the hypothalamus and pituitary gland and interplay with calcium to increase FSH levels (20, 21). Second, since lead may cross the blood-brain barrier, lead can restrain hypothalamic cells to secrete gonadotropin-releasing hormone (GnRH) and activate calmodulin (22, 23). Third, lead exposure can increase homocysteine levels, which can activate N-methyl-D-aspartate (NMDA) and γ-aminobutyric acid (GABA) (24–26). In turn, NMDA can promote FSH secretion and GABA can regulate GnRH secretion (27, 28). Therefore, that mentioned above supported the findings of this study. On the other hand, no significant associations of blood lead levels with BMD and the risk of fractures were observed, which was consistent with some previous studies (13). However, other studies found a significant relationship between BMD and blood lead levels (29, 30). A possible reason explaining this disparity was that blood lead levels have considerably decreased over the past three decades (30). Another reason may be that plasma lead was a better indicators of toxicity than blood lead levels (31). However, only blood lead levels were available in the NHANES.

This study found that women with elevated serum FSH levels had a lower BMD and a higher risk of fractures, which was consistent with previous study (32). It was documented that elevated FSH levels accelerated bone resorption and contributed to osteoclast formation, which was susceptive to the mitogen-activated protein kinase (MAPK), nuclear factor kappa B (NFκB), and akt pathways (33, 34). Furthermore, studies *in vitro* showed that FSH promoted bone resorption and bone loss by an isoform of FSH receptor on bone (33, 35, 36). A low BMD is a key predictor of fall and osteoporotic fracture (4). Therefore, it was rational that elevated serum FSH levels were linked to a lower BMD and a higher risk of fractures. Notably, further stratification analysis found that significant associations of serum FSH levels with lumbar spine BMD and the risk of fractures occurred only in postmenopausal women but not in premenopausal women. Some potential reasons would explain this phenomenon. First, rate of postmenopausal bone loss was higher than that of premenopausal women (4). Therefore, postmenopausal bone loss was more obvious and sensitive to FSH. Second, a recent study indicated that age-related bone loss began at about 39 years in the total femur but at 49 years in the lumbar spine (16). Thus, onset of bone loss in the lumbar spine was later than that in the total femur. Lumbar spine bone loss was trivial before the final menstrual period. Therefore, serum FSH levels affected lumbar spine BMD in postmenopausal women but not in premenopausal women. Third, serum FSH levels increased prior to the decrease in estradiol levels. Decrease in estradiol level occurred across the menopausal transition and was most obvious during the postmenopausal period (37, 38). Therefore, the impact of serum FSH levels on BMD was more considerable in postmenopausal women given the attenuated protective effect of estradiol levels on BMD.

## Strengths and limitations

The advantages of this study were as follows: First, data analyzed in this study were obtained from the NHANES III and the continuous NHANES 1999-2002, which are representative of the US population. Therefore, the findings of this study were accurate and can stand the test. Second, this was the first study to simultaneously examine the associations of serum FSH and blood lead levels with BMD and the risk of fractures. As a result, this study can provide additional evidence and suggestion for preventing bone loss in the later life of women. However, the limitations of this study should also be stated. First, estradiol levels were collected only in males aged 12 years or over and failed to be adjusted in this study. Second, this study was a cross-sectional study, which was poor in demonstrating the causal associations of serum FSH and blood lead levels with BMD and the risk of fractures. Furthermore, the impact of serum FSH levels on BMD may be hysteretic. Measurements of changes in serum FSH levels or dynamic serum FSH levels may be better to picture the association of serum FSH levels with BMD. Therefore, longitudinal studies are needed to confirm these associations in the future. Thirdly, this study focused on young women aged 35-60 years. It should be cautious when extrapolating the conclusion to older women.

In conclusion, lead exposure was significantly associated with serum FSH levels but not BMD and the risk of fractures. Elevated serum FSH levels were linked to a lower total femur BMD and a higher risk of fractures independently of blood lead levels and menopausal status. However, elevated serum FSH levels were associated with a lower lumbar spine BMD and a higher risk of fractures only in postmenopausal women but not in premenopausal women. Therefore, serum FSH levels should be used together with menopausal status to precisely prevent from bone loss at different sites in the later life of women.

## Data availability statement

The datasets presented in this study can be found in online repositories. The names of the repository/repositories and accession number(s) can be found below: https://www.cdc.gov/nchs/index.htm.

## Ethics statement

The studies involving human participants were reviewed and approved by the NHANES Institutional Review Board. The participants provided their written informed consent to participate in this study.

## Author contributions

HT contributed to writing the original draft. BS contributed to review and editing the draft. ZL contributed to formal analysis and results interpretation. YC contributed to study design and conceptualization. All authors contributed to the article and approved the submitted version.
